# MiR-145 functions as a tumor suppressor in Papillary Thyroid Cancer by inhibiting RAB5C

**DOI:** 10.7150/ijms.44723

**Published:** 2020-07-25

**Authors:** Wei Zhang, Wenyue Ji, Tianshu Li, Ting Liu, Xudong Zhao

**Affiliations:** 1 Department of Endocrinology, Shengjing Hospital of China Medical University, Shenyang, 110004, China.; 2 Department of Otolaryngology head and neck surgery, Shengjing Hospital of China Medical University, Shenyang, 110004, China.

**Keywords:** miR-145, RAB5C, papillary thyroid carcinoma, PTC

## Abstract

Papillary thyroid carcinoma (PTC) accounts for the largest proportion of thyroid cancers; and its morbidity rate has dramatically increased in recent decades. However, the pathogenesis mechanisms of PTC are still not clear. This study aimed to reveal that miR-145 acts as an antitumor miRNA in the progression of PTC. In the present study, the expression of miR-145 was analyzed in 57 paired PTC patient samples. The relationship between clinicopathological features and miR-145 expression were also defined. The tumor suppressive function of miR-145 on PTC cell metastasis, proliferation and apoptosis were revealed in vitro. Also, we used dual luciferase reporter assay to define the relationship of miR-145 and RAB5C. RAB5C was reported to participate in cell invasion and cell motility. We found that miR-145 was downregulated in PTCs, which was negatively correlated with PTC progression and metastasis. MiR-145 inhibited PTC migration, proliferation and promoted apoptosis by directly suppresing RAB5C. In conclusion, miR-145 functions as a tumor suppressor in PTC by inhibiting RAB5C. MiR-145 and RAB5C are potential therapeutic targets in therapy of aggressive PTC cases.

## Introduction

Thyroid carcinoma accounts for the most frequent malignancy of endocrine organs 1. Papillary thyroid carcinoma (PTC) accounts for almost 80% of all types of thyroid cancers and its morbidity rate has dramatically increased in recent decades 2, 3. Although PTC patients generally have a good prognosis, a small proportion of PTC patients have an aggressive form and are prone to further developing progressive disease 4, 5. Thus, it is necessary to reveal the cancer progression mechanism and find inhibitory factors.

MicroRNAs are small noncoding RNAs of 18-22 nucleotides which exert their regulatory functions through inhibition of translation or mRNA degradation 6-9. MicroRNAs can directly bind to the complementary 3'-untranslated region (3'-UTR) of mRNAs, which results in the inhibition of protein translation 10. MiR-145 was reported as a tumor suppressor of many solid tumors, such as pancreatic cancer 11, oral squamous cell cancer 12, laryngeal cancer 13, breast cancer 14-18, colorectal cancer 19-21 and gastric cancer 22-24. However, the function and mechanisms of miR-145 in PTC is still not clear.

Rab5 protein is a type of guanosine triphosphatase, which is related to endosomal classification and participates in the fusion of endosomal membrane 25. In humans, RAB5 family includes three isoforms (A, B, and C) sharing more than 90% of sequence identity. The three isoforms may have different functions 26-28. The RAB5C isoform was reported to participate in cell invasion and cell motility. Chen et al. found that RAB5C modulated Rac-mediated cell motility 29. Also, RAB5C was reported to regulate cell cohesion 30. Furthermore, RAB5C was shown to promote ovarian cancer and play important role in drug-resistance of ovarian cancer treatment 31. Tan et al. found that RAB5C was an important regulator in acute lymphoblastic leukemia 32. Also, Onodera et al. revealed that RAB5C promoted breast cancer invasion by AMAP1-PRKD2 complex 33. At present, the function of RAB5C in PTC remains largely not clear. The relationship of RAB5C and microRNAs was also reported by Tan et al. RAB5C was established as a target of miR-509 in the progression of acute lymphoblastic leukemia 32.

In current study, we reveal that miR-145 represses PTC progression by suppressing RAB5C for the first time.

## Materials and Methods

### Patient tissue samples and PTC cell line

Fifty-seven paired human PTC samples and adjacent healthy thyroid samples were acquired from patients in Shengjing Hospital of China Medical University between March 2015 and October 2016 (Supplementary Table 1). We acquired prior informed consent from all patients. The study protocol was approved by Shengjing Hospital Ethics Committee. Samples were obtained during surgical resections, immediately frozen in liquid nitrogen and stored at -80°C until use. The human papillary thyroid cancer cell line, BCPAP and K1, were obtained from the cell bank of Biochemistry and Cell Biology Institute of Chinese Academy of Sciences (Shanghai, China).

### Quantitative reverse-transcription polymerase chain reaction (qRT-PCR)

TRIzol reagent (Invitrogen, USA) was used to isolate total RNA from cells and clinical tissues. MiR-145 qRT-PCR assays were conducted by using an AmirVana^TM^ miRNA detection kit (Ambion, USA) and a Real-Time PCR system purchased from Applied biosystems, USA. Also, we used the 2^-ΔΔCT^ method to determine the relative miR-145 expression. We also used U6 as an endogen control. The reactions were performed following cycling protocol: 1 cycle at 95°C for 2min followed by 40 cycles of 95°C for 8 s and 60°C for 40 s.

CDNA synthesis was performed with the following primer:

- 5'-CTCAACTGGTGTCGTGGAGTCGGCAATTCAGTTGAGCTTTGGGA-3'.

The qRT-PCR primers for U6 were as follows:

- Forward, 5'-CTCGCTTCGGCAGCACATATACT-3';
- Reverse, 5'- ACGCTTCACGAATTTGCGTGTC.

The qRT-PCR primers for miR-145 were as follows:

- Forward, 5'-TCGGCAGGGTCCAGUUUUCCCAGG-3';
- Reverse, 5'-CAGTGCGTGTCGTGGAGT -3'.

### Western blotting

Tissues or BCPAP and K1 cells were firstly lysed by RIPA buffer (Beyotime, Shanghai, China) with 10 nM PMSF for 60 minutes and then extract total protein. Sodium dodecyl sulfate polyacrylamide gel electrophoresis (SDS-PAGE) was used to fractionate protein samples, and then proteins were transferred to PVDF membrane. The PVDF membrane was then blocked by 5% non-fat milk. After that, Rab5c or β-actin primary antibody (1: 1000, BD Technology, USA) were incubated with the PVDF membrane at 4°C overnight. After incubating with secondary antibody, UVP Image System (BD, USA) was used to detect the immunoreactive protein bands. Relative protein levels were quantified by Image J software.

### Cell culture and transfection

RPMI 1640 medium (Gibco, Grand Island, NY, USA) with 10% fetal bovine serum (FBS) purchased from Gibco, USA was used to culture BCPAP cells. Also, DMEM medium (Gibco, Grand Island, NY, USA) with 10% fetal bovine serum (FBS) purchased from Gibco, USA was used to culture K1 cells. Cells were cultured in their medium supplied with 10% FBS (fetal bovine serum) at 37°C in a humidified incubator containing 5% CO2. Two miR-145 mimics and a negative control were obteined from Cyagen Biosciences Inc. (China). RAB5C-specific siRNA and overexpression vector were also obtained from Cyagen Biosciences Inc. (China). Lipofectamine 2000^TM^ purchased from Invitrogen was used for cell transfection. Cells were transfected with miR-145 mimics or controls using Lipofectamine 2000™ according to the manufacturer's protocol. The miR-145 mimics transfection concentration was 50 nM.

### Cell proliferation assay

A Cell Counting Kit-8 (CCK-8) purchased from Beyotime was used to conduct the cell proliferation assay. Before inoculation, the first choice was the appliance, which used a 96-well plate on which BCPAP or K1 cells were inoculated at a density of 200 cells/Wells. Incubation was conducted after inoculation wherein 10 μl of CCK-8 reagent was added separately to 24, 48, 72 and 96 h incubation wells. A microplate reader purchased from Thermo Fisher (China) was used to measure the 450 nm absorbance.

### Cell migration assay

The migration of BCPAP or K1 cells was evaluated using Transwell assays (BD Biosciences, USA). After 24h transfection, we harvested and resuspended the BCPAP or K1 cells, and then seeded into the upper chambers at 4 × 10^4^ cells/well density. Then, 0.5 ml of DMEM or RPMI 1640 medium with 20% FBS was added to each chamber. The BCPAP or K1 cells were allowed to migrate for 24h at 37°C. Then, we used 4% paraformaldehyde to fix the migrated cells on the lower surface for 10 min. After that, crystal violet solution was used to stain the cells for 15 min at room temperature. Then we used a photomicroscope (BX51; Olympus, Japan) to image the BCPAP or K1 cells on lower surfaces.

### Luciferase reporter assay

BCPAP or K1 cells were cotransfected with RAB5C-3′-UTR-WT or Rab5c-MUT and miR-145 mimic or the negative control, using Lipofectamine 2000. BCPAP or K1 cells were transfected for 48 h. After that, Dual-Luciferase Reporter Assay System (Promega, CA, USA) was used for analysis. A GloMax fluorescence reader (Promega) was used to determine the luciferase activity. All experiments were repeated thrice.

### Flow cytometry and quantification of apoptotic cells

We seeded 0.3 × 10^6^ BCPAP or K1 cells each well and cultured overnight. Then we harvested and washed thrice using fluorescence-activated cell sorting buffer. The cells were incubated with Fc blocker at room temperature for 30 min. After that, the BCPAP or K1 cells were stained by Anti-Annexin V antibody (Abcam, USA) for 20 min at room temperature according to the manufacturer's protocol. Then we used an LSR II flow cytometer (BD Biosciences) to run the flow. Also, FlowJo software (Tree Star) was used to analyze the results. BCPAP or K1 cells treated with 0.1 mol/L 5-fluorouracil (5-FU) were used as positive control.

### Colorimetric caspase-3 assay

We lysed BCPAP or K1 cells and calculated the protein concentrations. One hundred microgram of proteins was treated with 10 μL of Ac-DEVD-pNA (Abcam, USA) and incubated at 37°C for 2 h. Then we used a microplate reader (Bio-Tek Instruments) to determine the 405 nm absorbance.

### Statistical analysis

All experiments were repeated for at least three times. We used the mean ± SD to summarize all the data. Also, we used SPSS 17.0 (SPSS Inc., USA) for all the statistical analyses. Firstly, all data were assessed by normal distribution test. And all the normally distributed data were analyzed by Student's *t*-test. Ratio results were log-transformed prior to Student's *t*-tests. *P* < 0.05 was considered statistically significant in all analyses.

## Results

### Downregulation of miR-145 in human PTC tissues

**Figure 1:**
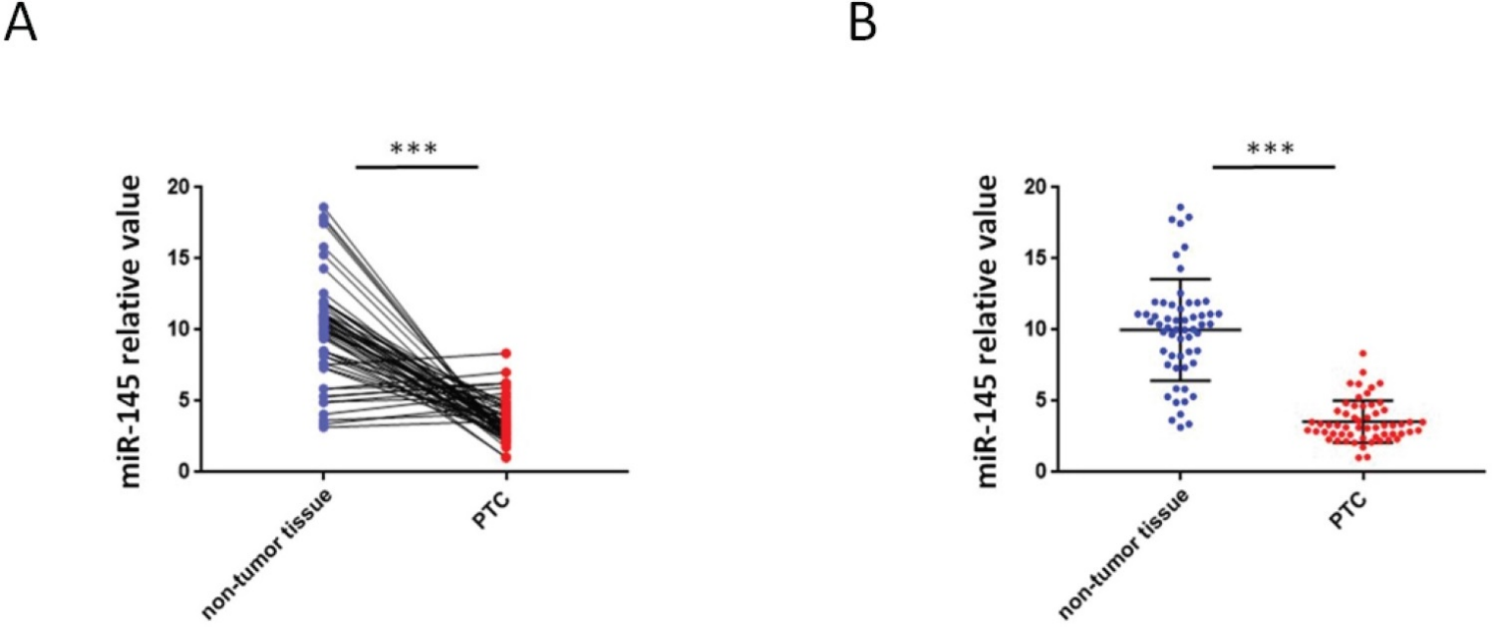
** Aberrant expression of miR-145 in PTC tissues**. (**A**) Quantification of the process of reverse transcription polymerase reaction (qRT-PCR) analysis of miR-145 expression in 57 paired PTC and adjacent non-tumor tissues. We found that 46/57 (80.7%) of PTC specimens had reduced miR-145 expression compared to adjacent non-malignant thyroid tissue (log-transformed of data was used prior to paired *t*-test analysis; *P* = 0.002). Each set of experiments was performed three times. The repetition of a creature is represented by a dot. (**B**) qRT-PCR analysis of miR-145 expression in PTC and adjacent normal tissue samples. Statistical analysis is carried out by t-test method, and the analysis is performed after logarithmic transformation. Each set of experiments was performed three times. The repetition of a creature is represented by a dot.

QRT-PCR were used to analyze the miR-145 expression in PTC and adjoining non-tumor tissue samples. We found that 46 PTC specimens had reduced miR-145 expression when comparing adjoining non-malignant thyroid tissue samples (Figure 1A) .Compared with adjoining normal tissues, PTC tissues exhibited less of the expression of miR-145. The mean relative expression level of miR-145 in total non-malignant thyroid tissue was 10.01 ± 3.56 and in the whole malignant sample 3.56 ± 1.46 (P = 0.005). Therefore, compared to adjacent non-malignant thyroid tissue, PTC cases exhibited about 2.81-twice lower level of miR-145 expression (Figure 1B).

### MiR-145 act as a suppresser in PTC progression and metastasis

The clinical significance of miR-145 in human PTC is still unclear. The clinicopathological parameters in patient records of the 57 patients were reviewed. Tumor size, age, extrathyroidal invasion, sex, cervical lymph node metastasis and multicentricity were analyzed for associations with the expression of miR-145 (Table 1). There were no significant associations between the expression of miR-145 and age, sex, tumor size, multicentricity, extrathyroidal invasion. But the expression of miR-145 was significantly upregulated in patients in the early-TNM stage and without cervical lymph node metastasis. Collectively, these results suggested that miR-145 act as a suppressor in human PTC progression and metastasis.

### Enhanced miR-145 expression suppresses proliferation and migration, and promotes apoptosis of BCPAP and K1 cells *in vitro*

**Figure 2:**
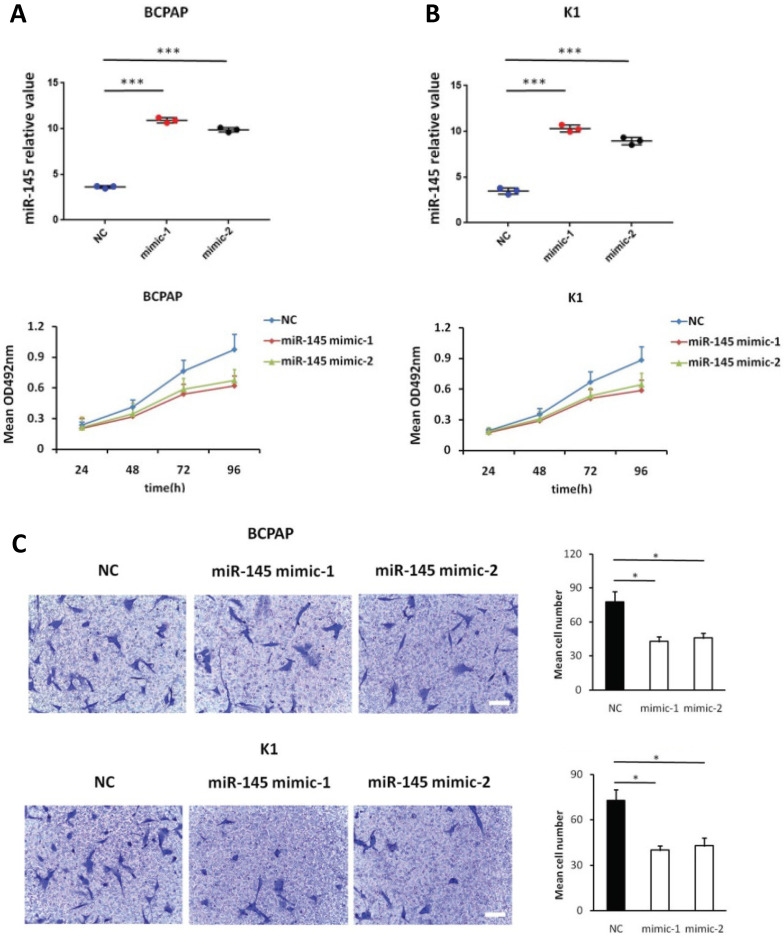

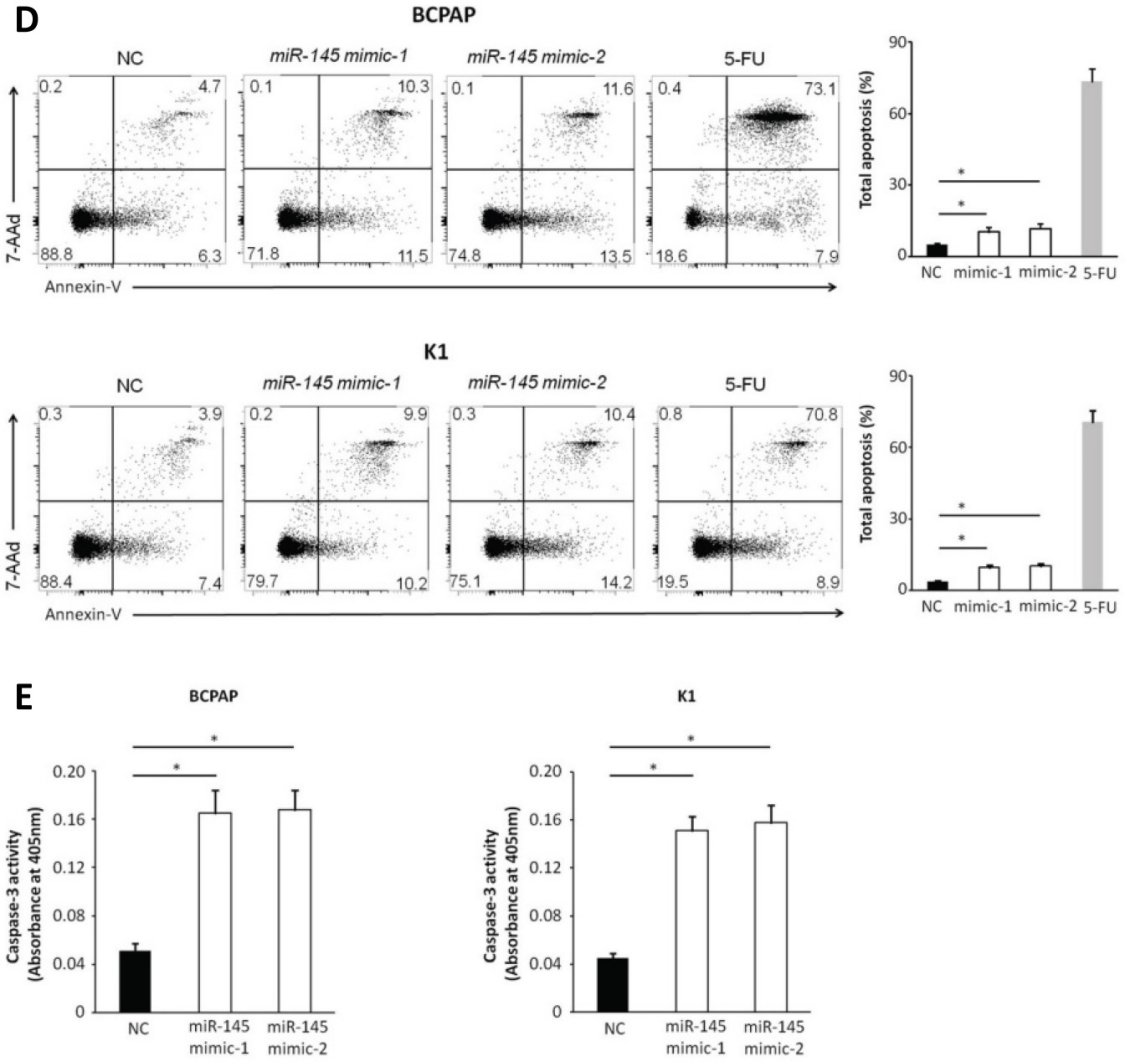
** Effect of miR-145 on PTC cell proliferation, migration, and apoptosis.** (**A**) BCPAP and K1 cells were transiently transfected with two mimics of miR-145 or negative control (*P* < 0.001). (**B**) Cell survival rates determined by MTT assays in miR-145-overexpressed and control BCPAP and K1 cells. (**C**) Representative photographs of Transwell aggressive attacks on miR-145 overexpression and negative effects on BCPAP and K1 cells (upper) and their quantitative analysis (bottom). Scale bar, 50 µm. (*represents *P* < 0.05). (**D**) Representative photographs and proportion of apoptosis of BCPAP and K1 cells with miR-145-overexpression or negative control (NC) and positive control (treated with 5-FU) (* represents *P* < 0.05). (**E**) Caspase-3 activity in BCPAP and K1 cells transfected with the mimics of miR-145 or negative control (* represents *P* < 0.05). Each set of experiments was performed three times. The repetition of a creature is represented by a dot.

We used MTT assays to determine the influence of miR-145 overexpression on the proliferation of BCPAP and K1 cells. We transiently transfected BCPAP and K1 cells with miR-145 mimics or negative control (Figure 2A). QPCR was used to identify the expression of miR-145 in each (BCPAP cells: 10.91 ± 0.17 and 9.87 ± 0.25 vs. 3.61 ± 0.08 [control], *P* < 0.001; K1 cells: 10.31 ± 0.22 and 8.94 ± 0.41 vs. 3.48 ± 0.02 [control], *P* < 0.001). MTT assays demonstrated that transfection with miR-145 mimic suppressed the growth of BCPAP and K1 cells comparing with the negative control in a time-dependent manner. Decline with time was found in the cell rate of increase in cell number of the miR-145-overexpression cells (Figure 2B). To determine the migration potential of BCPAP and K1 cells overexpressing miR-145, we used Transwell assays. MiR-145 overexpression resulted in decreased BCPAP and K1 cell migration comparing with negative control (Figure 2C). Next, Annexin-V staining was used to determine the influence of miR-145 on PTC apoptosis. Overexpression of miR-145 significantly enhanced BCPAP and K1 cell apoptosis (Figure 2D). Also, similar results were obtained using colorimetric caspase 3 assay to evaluate BCPAP and K1 cell apoptosis (Figure 2E). Taken together, these data clearly indicated that miR-145 can inhibit PTC cell migration and proliferation, also promotes apoptosis.

### MiR-145 can directly inhibit RAB5C expression in PTC

**Figure 3:**
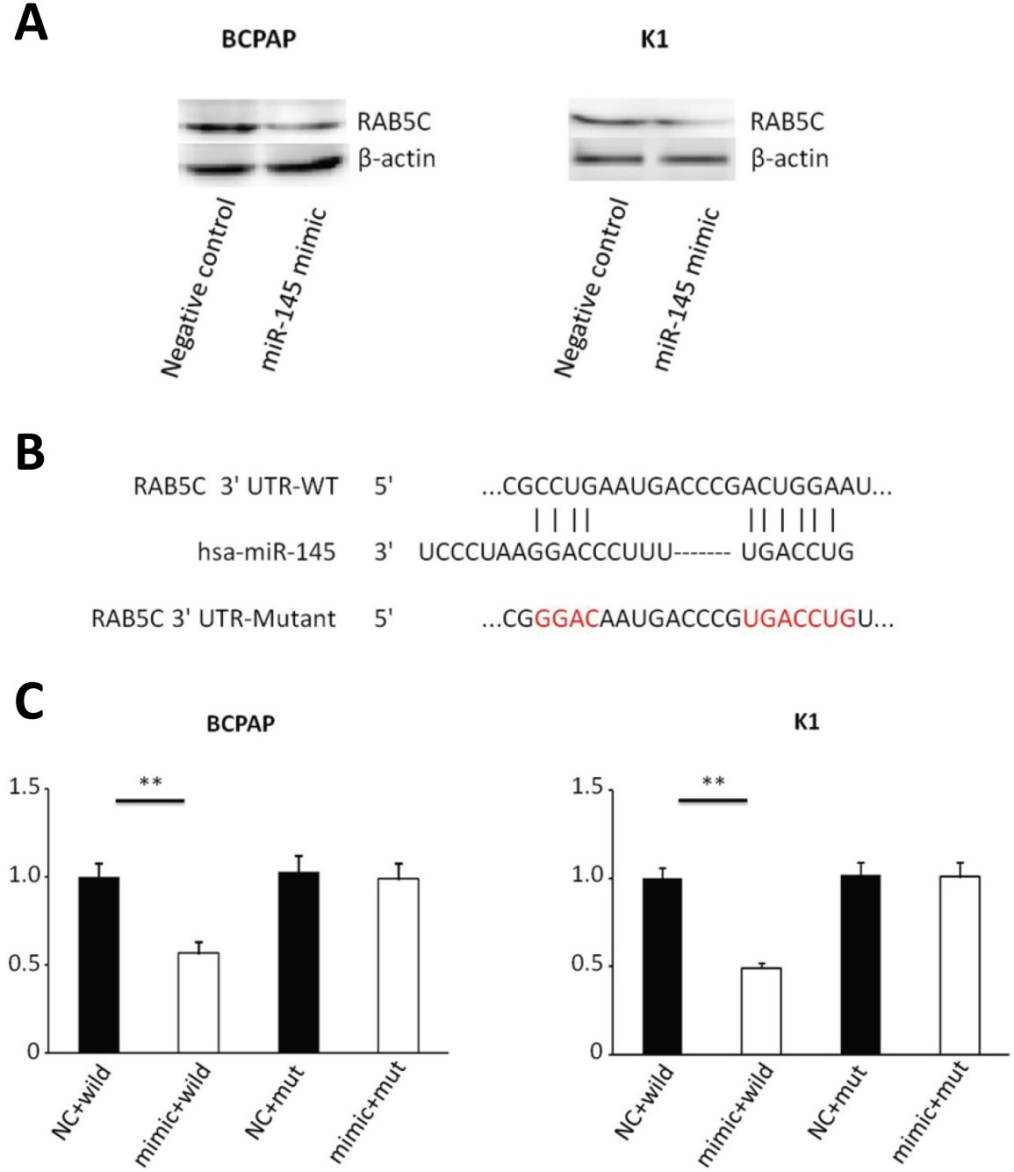
** RAB5C is a direct target of miR-145 in PTC.** (**A**) Analysis of representative western blot and RAB5C proteins by miR-145 simulation or negative comparison analysis of BCPAP and K1 cells (*P* = 0.031 and *P* = 0.040). β-actin was used as an endogen control. (**B**) miR-145 straight from the shoulder interacted with the 3′-UTRs of RAB5C. (**C**) After co-transfection with miR-145 mimic, the relative luciferase activity of the reporter plasmid containing the uncultivated-type RAB5C 3'-UTR was significantly reduced. But the miR-145 level change did not produce an effect on the luciferase activity of the reporter plasmid with the RAB5C mutant 3'-UTRs.These data are the mean ± SD of the results of three trials that do not interfere with each other. After the logarithmic transformation of the data by two-way analysis of variance, it is statistically organized and analyzed. **P = 0.022 and 0.015 for BCPAP and K1 cells, respectively. Dots represent experimental repetitions.

Bioinformatics analysis (miRWalk, TargetScan, and PicTar) predicted that RAB5C is a direct target of miR‑145. We determined the relationship between miR-145 and RAB5C in K1 and BCPAP cells. MiR-145 mimic-mediated miR-145 overexpression was associated with a significant decrease in RAB5C expression comparing with the negative control. RAB5C relative expression was suppressed 62.8% in BCPAP cells and 66.2% in K1 cells separately by miR-145 overexpression (Figure 3A). We determined the association of miR-145 and RAB5C by using luciferase reporter assays. Firstly, we constructed luciferase reporter plasmids which contains a mutant or the wild-type 3′-UTR of RAB5C (Figure 3B). We found significant decrease of luciferase activity of wild-type RAB5C 3′-UTR reporter upon cotransfection with miR-145 mimic, whereas that of the mutant 3′-UTR reporter was unaffected by cotransfection with miR-145 mimic (Figure 3C). Collectively, these suggested that miR-145 can directly inhibit RAB5C in PTC.

### Forced RAB5C overexpression restores the inhibitory effects of miR-145

**Figure 4:**
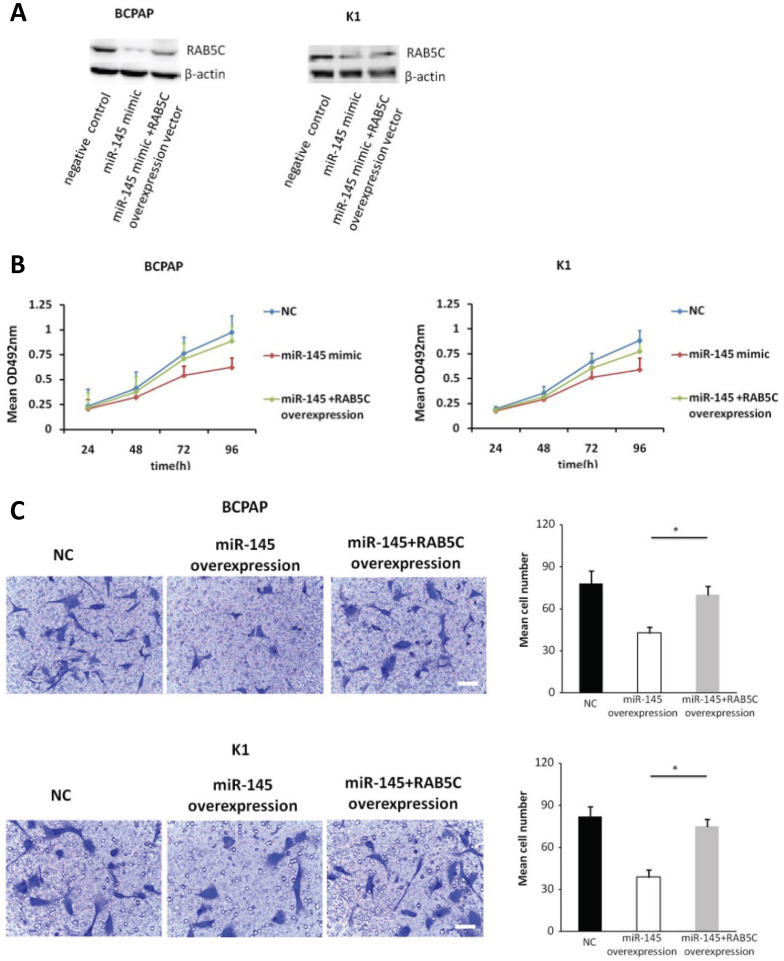

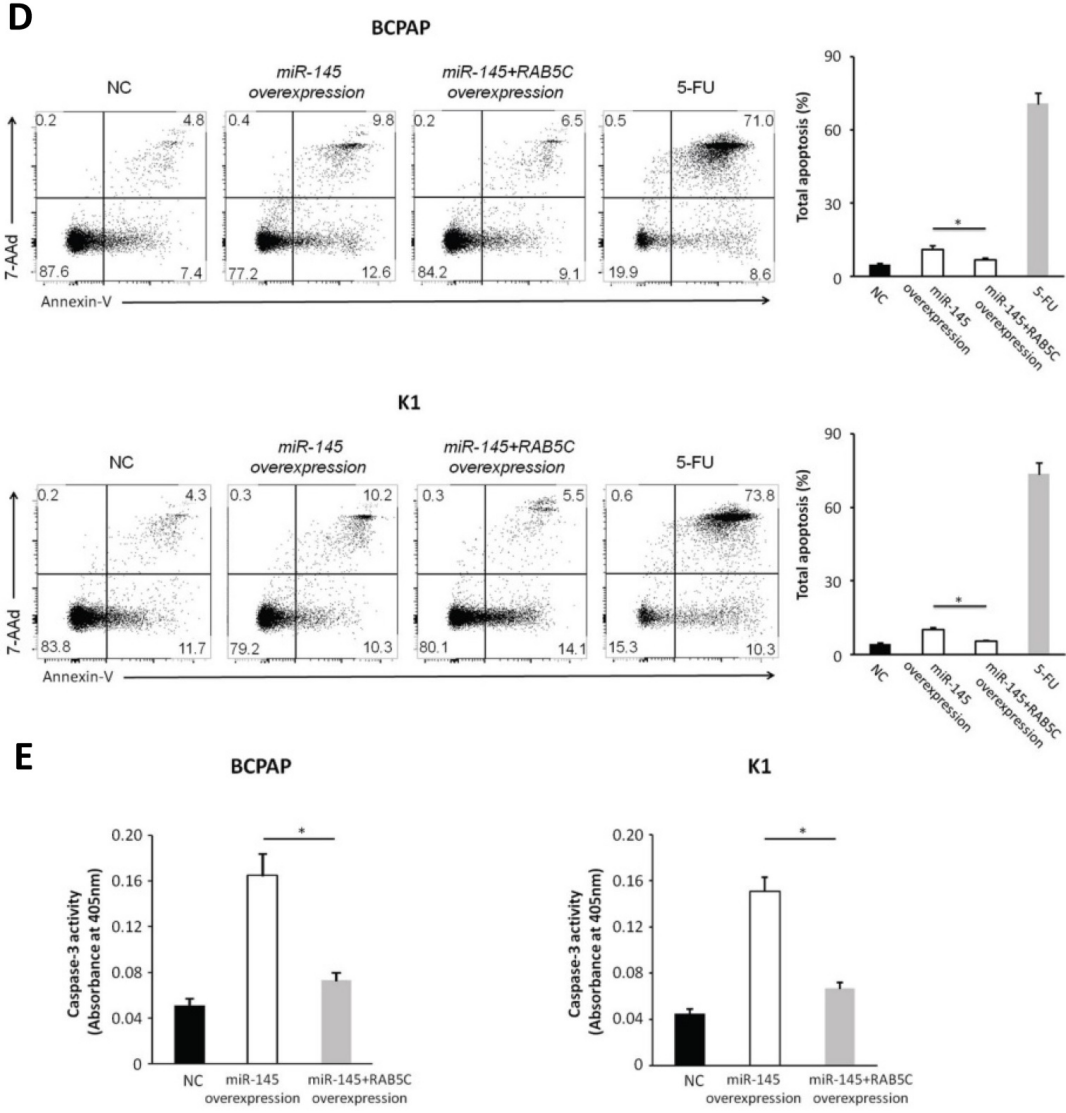
** RAB5C overexpression restores the inhibitory effects of miR-145.** (**A**) Typical western blot findings delivery that transfection of RAB5C-overexpression carrier comtrastto restores the depression of RAB5C after transfection with miR-145 mimic. (**B**) Value added rates of miR-145-overexpression BCPAP and K1 cells at different time points after RAB5C overexpression. (**C**) Typical pictures (upper) of Transwell aggressive attacks of miR-145-overexpressing BCPAP and K1 cells with or without RAB5C overexpression and analysis of specific quantities (bottom) Scale bar, 50 µm. (P = 0.035 and P = 0.023). (**D**) Typical pictures showing the ratio of BCPAP and K1 cells in apoptosis after transfection with miR-145 mimic with and without RAB5C overexpression (P = 0.036 and 0.031). (**E**) Caspase-3 doings of BCPAP and K1 cells transfection with miR-145 mimic have and without RAB5C over-deliverance (P = 0.029 and 0.018). NC: negative control.

In order to explore the inhibitory effects of miR-145 targeting RAB5C in PTC, a RAB5C overexpression vector was transfected into BCPAP cells and K1 cells with miR-145 overexpression (Figure 4A) to restore RAB5C expression (BCPAP cells: 49.88 ± 4.10 vs. 29.75 ± 2.41 transfection with mimic alone, *P* = 0.042; K1 cells: 55.52 ± 5.03 vs. 20.33 ± 3.17 transfection with mimic alone, *P* = 0.036). We observed a time-dependent increased proliferation in BCPAP cells with RAB5C rescue, comparing with miR-145 overexpression only (6.3 ± 0.3, 16.7 ± 2.3, 31.2 ± 3.8, and 42.6 ± 4.9% at 24h, 48h, 72h, and 96h, respectively, *P* = 0.021) ; Also, the rescue effect in K1 cells was also observed (4.5 ± 0.5, 8.2 ± 1.9, 18.4 ± 3.1, and 31.7 ± 3.8% at 24h, 48h, 72h, and 96 h, respectively, *P* = 0.039) (Figure 4B). Comparing with miR-145-overexpressing BCPAP and K1 cells, overexpression of RAB5C restored the inhibitive effects of miR-145 on migration and proliferation (Figure 4C). Moreover, overexpression of RAB5C significantly inhibited apoptosis of BCPAP and K1 cells (Figure 4D and 4E). Taken together, these findings suggested that miR-145 suppresses human PTC progression via RAB5C.

## Discussion

In endocrine system, thyroid cancer is the most common, and the incidence has increased in past decades. PTC accounts for the most common portion in all the thyroid cancer 34. Most PTC patients can be effectively treated with surgery; however, 10%-15% of patients with PTC have poor prognosis 35. Therefore, the identification of potential targets which can inhibit the progression in PTC is urgently needed.

The miR-145 gene is found located on chromosome 5 (5q32-33) 36. MiR-145 reportedly acts as an inhibitor in numerous solid malignancies. Azmi et al. highlighted miR-145 as a tumor suppressor in pancreas 11. Rodrigues et al. reported that miR-145 inhibits oral squamous cell carcinoma progression 12. Devlin et al. revealed that miR-145 has anticancer effects in breast cancer 15. Hu et al. revealed that miR-145 inhibited the progression of gastric cancer 22. Moreover, several researches have revealed that miR-145 is a novel biomarker of various solid malignancies 19, 21, 24. In this study, we found that the expression of miR-145 was decreased in 46 out of 57 PTC tissues evaluated. Eighty percent of the PTC showed decreased miR-145 expression. That means the expression of miR-145 was generally decreased in PTC. After that, in order to determine the clinical significance of miR-145, we analyzed potential correlations between miR-145 and clinicopathological factors. We found that lower miR-145 expression associated with advanced stages and the presence of cervical metastasis. So we believe that the lower miR-145 expression may promote cervical metastasis in clinical practice. Also, patients with large tumor size showed lowered miR-145 expression, but this finding is not statistically significant.

After that, BCPAP and K1 cells were used to reveal the effect and mechanism of aberrant miR-145 expression in PTC. The online tools were used to predict the targets of miR-145. Then, RAB5C was identified as a potential target of miR-145. RAB5C is a type of guanosine triphosphatase which participate in endosomal membrane fusion reactions and can regulate sorting endosome 25. Jin et al. reported aberrant expression of RAB5C in ovarian cancer 31. Moreover, Onodera et al. revealed that RAB5C promoted invasion of breast cancer cells 33. In our present study, we increased miR-145 expression in cell lines. And we found the proliferation and invasion of cells were inhibited and apoptosis increased significantly. These results confirmed miR-145 can inhibit the progression of PTC, the same as previous conclusions in clinical data. Also, BCPAP and K1 cells were used to reveal the correlation between miR-145 and RAB5C. MiR-145 overexpression was correlated with significant inhibition of RAB5C expression. Luciferase reporter assays were used to show that miR-145 can directly bind to the 3′-UTR of RAB5C, affecting the RAB5C expression. These results indicated that RAB5C is target of miR-145 in PTC. Moreover, we found that overexpression of RAB5C reversed the effects of miR-145 overexpression on cell migration and proliferation, and apoptosis. These results revealed that miR-145 can inhibit the PTC progression via RAB5C. But in our results we found that RAB5C reversed the effects of miR-145 overexpression partially, not completely. So we speculated there were other downstream targets of miR-145 in PTC. Our further study will focus on this.

Our study had some limitations. Namely, further research of tumorigenicity assays on nude mice is needed to show the role of miR-145 and RAB5C in PTC progression.

Conclusively, our study revealed that aberrant miR-145 expression affects PTC progression: miR-145 inhibition causes RAB5C overexpression and cancer progression. Therefore, miR-145 and RAB5C could be potential therapeutic targets in the therapy of aggressive PTC cases.

## Supplementary Material

Supplementary table.

## Figures and Tables

**Table 1:** Correlation of miR-145 expression with clinicopathological factors of PTC patients

Parameters	Patients	miR-145 relative expression	*P*
**Total gender**	57		0.641
Male	14 (24.6)	3.71±1.54	
Female	43 (75.4)	3.48±1.43	
**Age (Y)**			0.213
≥45	33 (57.9)	3.37±1.57	
< 45	24 (42.1)	3.83±1.34	
**Tumor size, cm**			0.097
≥2	25 (43.9)	3.29±1.75	
< 2	32 (56.1)	3.85±1.09	
**Extrathyroidal invasion**			0.116
Yes	13 (22.8)	3.38±2.02	
No	44 (77.2)	3.95±1.17	
**Multicentricity**			0.437
Yes	18 (31.6)	3.40±1.24	
No	39 (68.4)	3.89±1.57	
**Cervical metastasis status**			0.022
N+	23 (45.6)	2.87±1.60	
N-	34 (54.4)	4.54±1.37	
**TNM stages**			0.041
I/II	31 (54.4)	4.21±1.26	
III/IV	26 (45.6)	2.71±1.71	

Note: Values in parentheses represent percentages.
