# Supplementary material for: MiR-145 functions as a tumor suppressor in Papillary Thyroid Cancer by inhibiting RAB5C
Source: Int J Med Sci. 2020 Jul 25;17(13):1992–2001. doi: 10.7150/ijms.44723 (PMC7415399; doi:10.7150/ijms.44723)
Supplement: Supplementary file 1 — Supplementary table. [file ijmsv17p1992s1.pdf]

Supplementary Table 1. Information of PTC patients

No.	gender	age	tumor size	Extrathyroidal invasion	Multicentricity	Cervical metastasis status
1	female	41	1.8	−	−	N0
2	female	33	1.6	−	−	N0
3	male	55	3.2	+	+	N1a
4	female	40	1.1	−	−	N0
5	female	65	4.1	+	+	N1a
6	female	29	1.9	−	−	N0
7	female	44	1.8	−	−	N0
8	female	40	1.8	−	−	N0
9	male	58	2.3	−	−	N0
10	male	46	1.5	−	−	N0
11	female	56	3.6	+	+	N1b
12	female	63	2.8	−	−	N0
13	female	37	1.7	−	−	N0
14	female	69	3.7	+	+	N1a
15	female	43	1.9	−	−	N0
16	female	40	1.6	−	−	N0
17	female	75	3	−	+	N1a
18	female	41	1.8	−	−	N0
19	male	64	2.6	−	−	N1a
20	male	42	1.9	−	−	N0
21	female	58	3.3	−	+	N1b
22	male	48	1.5	−	+	N1a
23	female	39	1	−	−	N0
24	female	36	1.2	−	−	N0
25	male	71	4.5	+	+	N1a
26	female	80	4.6	+	−	N1a
27	female	64	1.8	−	−	N0
28	female	42	1	−	−	N1a
29	female	51	1.6	−	−	N0
30	male	69	5.6	+	+	N1a
31	female	32	1.1	−	−	N0
32	male	63	6	+	−	N1b
33	female	47	1	−	−	N0
34	female	69	4.4	+	+	N1b
35	female	35	1.9	−	−	N0
36	female	77	2.4	−	−	N1a
37	female	41	1.5	−	−	N0
38	female	74	1.2	−	+	N0
39	male	49	1.8	−	−	N0
40	female	68	2.6	−	+	N1a

41	female	43	1.8	−	−	N0
42	female	71	2.8	−	−	N0
43	female	42	1	−	−	N0
44	male	59	3.5	−	+	N1a
45	female	63	5.1	+	+	N1b
46	female	40	1.5	−	−	N0
47	male	75	2.2	−	−	N0
48	female	66	2.9	−	−	N1a
49	male	42	1.4	−	+	N0
50	female	74	0.9	−	−	N1a
51	male	60	5.5	+	+	N1b
52	female	41	1.8	−	−	N0
53	female	57	2.4	−	−	N0
54	female	38	1.9	−	−	N0
55	female	61	4.1	+	−	N1a
56	female	44	1.6	−	+	N0
57	female	76	3.9	+	+	N1b
